# An Underrecognized Problem: Missed and Delayed Carbidopa‐Levodopa Administration in Emergency Department Patients With Parkinson's Disease

**DOI:** 10.1111/acem.70269

**Published:** 2026-03-30

**Authors:** Natalie M. Elder, Erica M. Lash, Christopher Tomkins‐Tinch

**Affiliations:** ^1^ Department of Emergency Medicine The Robert Larner College of Medicine at the University of Vermont Burlington Vermont USA; ^2^ Independent Researcher

## Abstract

**Introduction:**

Patients with Parkinson's disease (PD) frequently present to the Emergency Department (ED). Whether for PD‐related complications or unrelated concerns, maintaining their antiparkinsonian medication regimen without interruption is crucial. Delays or omissions can lead to significant morbidity and mortality. Despite this, the importance of timely ordering and administration of antiparkinsonian medications is often underrecognized in the ED.

**Methods:**

We performed a retrospective chart review across a single health system comprising one academic and five community EDs, three of which are critical access hospitals. Adults aged ≥ 65 years with an active outpatient carbidopa‐levodopa (C‐L) prescription presenting between September 1, 2024, and August 31, 2025, were included. The primary outcome was the proportion of patients who received their prescribed C‐L during the ED encounter. Timeliness was assessed using two definitions: a primary, idealized standard of administration within 30 min of the scheduled dose, and a secondary, system‐based standard of administration within a two‐hour window.

**Results:**

A total of 282 patient encounters involving 87 unique patients were included (mean age 80.1 years; 61.7% male; 99.3% White). Mean ED length of stay (LOS) was 8 h and 53 min. C‐L was administered in only 91 encounters (32.3%). Among the 282 ED encounters, 12 (4.3%) met the idealized timeliness definition for C‐L administration, and 18 (6.4%) met the system‐defined standard. Among the 91 encounters with a C‐L order, 13.2% met the ideal definition and 19.8% met the system standard. Mean time from ED arrival to medication administration was 6 h 11 min. Most administrations occurred 1–4 h (39.6%) or 4–8 h (26.4%) after the scheduled dose.

**Conclusion:**

Less than one‐third of older adults with PD received their home antiparkinsonian medication in the ED, and fewer than 10% received it on time. Targeted interventions to ensure timely medication administration are needed to prevent iatrogenic harm in this vulnerable population.

## 1 Introduction

Parkinson's disease (PD) is a chronic, progressive movement disorder affecting more than 6 million people worldwide and represents the fastest‐growing neurologic disease in prevalence, disability, and mortality [1]. Patients with PD are frequently encountered in the Emergency Department (ED). Compared to age‐matched cohorts, those with PD experience higher rates of ED visits and hospitalizations, longer lengths of stay, increased morbidity and mortality, higher rates of recurrent hospital encounters, greater healthcare costs, and reduced quality of life [1, 2, 3, 4].

Whether patients with PD present to the ED for a PD‐related emergency or an unrelated reason, continuation of home dopaminergic medications is critical. Timely dosing maintains stable dopamine levels in the brain, thereby controlling motor symptoms [5]. In inpatient and ED settings, delayed administration of dopaminergic medications can result in significant complications [6, 7]. Delays may worsen motor symptoms including rigidity, tremor, bradykinesia, and freezing of gait, which can contribute to immobility, falls, aspiration, and pressure injuries. Delayed administration can also induce neuropsychiatric complications, including agitation, anxiety, confusion, delirium, and hallucinations or psychosis. Autonomic dysfunction may be exacerbated, increasing the risk of orthostatic hypotension, urinary retention, and constipation. Additionally, abrupt withdrawal of antiparkinsonian medications can precipitate Parkinsonism‐hyperpyrexia syndrome, a rare but life‐threatening condition [8]. Despite the significant morbidity and potential mortality associated with delayed or withheld doses, the importance of timely ordering and administration of antiparkinsonian medications is often underrecognized in the ED [7].

The goal of this retrospective chart review is to assess the frequency and timeliness with which patients with PD are administered their home antiparkinsonian medication while in the ED. This study focuses on patients prescribed carbidopa‐levodopa (C‐L), the most common dopaminergic medication prescribed for managing PD symptoms.

## 2 Methods

### 2.1 Study Design and Setting

We conducted a retrospective chart review of patients presenting to six EDs within a single health system who were prescribed C‐L at the time of their ED encounter. Data collection focused on ED encounters occurring between September 1, 2024 and August 31, 2025. The health system includes one academic ED (ED1), and five community EDs (EDs 2–6), three of which are designated as critical access hospitals (EDs 4–6). All six EDs have an automated medication dispensing cabinet stocked with C‐L within the department. The academic ED and one of the community EDs have ED pharmacists present during the daytime only. There is no formal medication reconciliation process at 5 of the 6 EDs (including the academic site). The pharmacists at ED 2, an urban community ED, routinely perform medication reconciliations on patients in the ED who will be admitted and at the request of the ED provider or consultant. The study was approved by the institutional review board with a waiver of informed consent due to the retrospective nature of the review.

### 2.2 Study Population

Older adults (≥ 65 years) presenting to the ED with an active outpatient prescription for C‐L were included in the study cohort.

### 2.3 Study Protocol

Electronic medical records (EMRs) were queried to identify ED encounters between September 1, 2024 and August 31, 2025 using the search terms “carbidopa‐levodopa,” and “emergency department visits.” Each ED encounter was analyzed as an independent observation, including repeat visits by the same patient, as encounters were considered clinically distinct due to limited continuity of care, differing presenting complaints, and variability in treating teams across visits.

All chart abstractions were performed by a single physician investigator, ensuring a consistent approach to data collection and interpretation across sites. Prior to data collection, study objectives and variable definitions were clearly defined. Variables were specified using explicit operational definitions, including criteria for determining outpatient C‐L dosing schedules, medication ordering, and timing of medication administration.

Data were abstracted from predefined sections of the electronic medical record, including ED provider documentation, outpatient medication lists, prescription records, pharmacy fill history when available, ED timelines, and medication administration records (MAR). For encounters with incomplete or missing data, abstraction was limited to information explicitly documented in the medical record. For outpatient C‐L prescriptions with unspecified administration times, dosing times were assigned using standardized EMR default scheduling parameters, as described above.

Interrater reliability was not assessed, as all abstractions were completed by a single reviewer.

### 2.4 Measurements and Key Outcomes

The primary outcome was the proportion of patients with PD prescribed outpatient C‐L who were administered this medication during their ED encounter.

Medication‐related timing and adherence variables included: whether C‐L was ordered while the patient was physically in the ED, ordering specialty, and timing of the administered dose relative to its scheduled time based on the patient's outpatient C‐L prescription. Timeliness of administration was assessed using two definitions. The primary definition reflected an idealized standard of administration within 30 min of the scheduled dose, consistent with prior inpatient literature. A secondary, system‐based definition reflected institutional medication administration standards, defined as administration within a two‐hour window of the scheduled time (up to one hour before or one hour after the scheduled dose). For outpatient prescriptions with unspecified administration times, dosing times were assigned based on the default EMR scheduling parameters (Table A1). These parameters represent the administration times automatically generated in the EMR when a medication order is entered with a specified frequency. For example, a three‐times‐daily (TID) regimen defaults to administration at 9:00, 14:00, and 21:00.

Additional variables collected included patient age, sex, race/ethnicity, ED location, ED length of stay (LOS), and disposition.

### 2.5 Data Analysis

Data were analyzed using Microsoft Excel. Descriptive statistics included means, medians, standard deviations, counts, and percentages.

## 3 Results

Between September 1, 2024 and August 31, 2025, there were 283 ED encounters involving 87 patients who had an active outpatient prescription for C‐L at the time of their ED visit. One encounter was excluded because the patient died shortly after ED arrival. Three patients were transferred from critical access EDs (EDs 4–6) to higher‐level care sites (EDs 1 or 2); both the initial and receiving ED encounters were included as separate observations. An additional three patients were transferred to facilities outside the health system; only their initial ED encounter within the system was included in the analysis. Therefore, 282 ED encounters corresponding to 87 unique patients were analyzed.

Six EDs within the health system were included in the analysis. The largest proportion of patient encounters occurred at ED1, a tertiary academic medical center (*n* = 97, 34.4%). ED2, an urban community hospital, accounted for 61 encounters (21.6%), and ED3, a rural community site, for 37 encounters (13.1%). The remaining 87 encounters (30.9%) occurred at ED4 (*n* = 45), ED5 (*n* = 21), and ED6 (*n* = 21), all of which are rural critical access hospitals.

The mean age at encounter was 80.1 ± 6.9 years, 61.7% were male, 99.3% were White, and 98.9% identified as not Hispanic, Latino/a, or Spanish origin. With respect to disposition, 40.4% were admitted, 57.4% discharged, and 2.1% were transferred (Table 1). The average ED length of stay (LOS) was 8 h 53 min (SD 9 h 12 min), with a median ED LOS of 5 h 54 min.

**TABLE 1:** Baseline characteristics of ED patient encounters.

Characteristic	ED1‐ Tertiary Academic Medical Center (*n* = 97)	ED2‐urban community (*n* = 61)	ED3‐rural community (*n* = 37)	ED4‐rural community critical access (*n* = 45)	ED5‐rural community critical access (*n* = 21)	ED6‐rural community critical access (*n* = 21)	Total (*n* = 282)
Age at encounter, mean (SD)	79.1 (5.6)	83.3 (5.9)	82.1 (4.7)	78.9 (6.9)	78.4 (12)	76.3 (7.8)	80.1 (6.9)
Age group, *n* (%)							
65–74 years	18 (18.6)	4 (6.6)	0 (0)	15 (33.3)	9 (42.9)	16 (7.6)	62 (22.0)
75–84 years	64 (65.9)	33 (54.1)	22 (59.5)	11 (24.4)	3 (14.2)	0 (0)	133 (47.2)
≥ 85 years	15 (15.5)	24 (39.3)	15 (40.5)	19 (42.2)	9 (42.9)	5 (2.4)	87 (30.8)
Gender, *n* (%)							
M	60 (61.9)	34 (55.7)	20 (54.1)	25 (55.6)	15 (71.4)	20 (95.2)	174 (61.7)
F	37 (38.1)	27 (44.3)	17 (45.9)	20 (44.4)	6 (28.6)	1 (4.8)	108 (38.3)
Race, *n* (%)							
White	95 (97.9)	61 (100)	37 (100)	45 (100)	21 (100)	21 (100)	280 (99.3)
Patient declines to respond	2 (2.1)	0 (0)	0 (0)	0 (0)	0 (0)	0 (0)	2 (0.7)
Ethnicity, *n* (%)							
Not Hispanic, Latino/a, or Spanish origin	94 (96.9)	61 (100)	37 (100)	45 (100)	21 (100)	21 (100)	279 (98.9)
Patient declines to respond	3 (3.1)	0 (0)	0 (0)	0 (0)	0 (0)	0 (0)	3 (1.1)
Disposition, *n* (%)							
Admit	41 (42.3)	25 (41.0)	19 (51.4)	13 (28.9)	6 (28.5)	10 (47.6)	114 (40.4)
Discharge	55 (56.7)	34 (55.7)	18 (48.6)	31 (68.9)	14 (66.7)	10 (47.6)	162 (57.4)
Transfer	1 (1.0)	2 (3.3)	0 (0)	1 (2.2)	1 (4.8)	1 (4.8)	6 (2.1)

Among the 282 patient encounters, C‐L was administered in 91 encounters (32.3%). Administration of C‐L was most frequent at ED1 (*n* = 43, 44.3%), and ED2 (*n* = 25, 41%), and less common at ED3 (*n* = 10, 27%), ED4 (*n* = 10, 22.2%), and ED5 (*n* = 3, 14.3%). No patients at ED6 received C‐L. Table 2 contains data for C‐L administration encounters. Ordering specialties included emergency medicine (64.8%), internal medicine (28.6%), neurology (4.4%), and general surgery (2.2%). The mean time from ED arrival to medication administration was 6 h 11 min (SD 4 h 30 min), with a median of 5 h 18 min.

**TABLE 2:** Characteristics of patient encounters in which C‐L was administered.

	ED1‐Tertiary Academic Medical Center (*n* = 43)	ED2‐urban community (*n* = 25)	ED3‐rural community (*n* = 10)	ED4‐rural community critical access (*n* = 10)	ED5‐rural community critical access (*n* = 3)	ED6‐rural community critical access (*n* = 0)	Total (*n* = 91)
Age at encounter, mean (SD)	79.3 (5.61)	84.0 (6.7)	85.4 (2.9)	79.2 (7.2)	76.7 (12.6)	N/A	81.2 (6.6)
Age group, *n* (%)							
65–74 years	7 (16.3)	1 (4.0)	0 (0)	3 (30)	1 (33.3)	N/A	12 (13.2)
75–84 years	28 (65.1)	13 (52.0)	8 (80)	3 (30)	1 (33.3)	N/A	53 (58.2)
≥ 85 years	8 (18.6)	11 (44.0)	2 (20)	4 (40)	1 (33.3)	N/A	26 (28.6)
Gender, *n* (%)							
M	28 (65.1)	17 (68.0)	4 (40)	7 (70)	1 (33.3)	N/A	57 (62.6)
F	15 (34.9)	8 (32.0)	6 (60)	3 (30)	2 (66.7)	N/A	34 (37.4)
Disposition, *n* (%)							
Admit	24 (55.8)	18 (72.0)	7 (70)	5 (50)	1 (33.3)	N/A	55 (60.4)
Discharge	19 (44.2)	7 (28.0)	3 (30)	4 (40)	1 (33.3)	N/A	34 (37.4)
Transfer	0 (0)	0 (0)	0 (0)	1 (10)	1 (33.3)	N/A	2 (2.2)
C‐L Prescription Frequency, *n* (%)							
BID	1 (2.3)	0 (0)	0 (0)	0 (0)	0 (0)	N/A	1 (1.1)
TID	21 (48.9)	18 (72)	5 (50)	1 (10)	1 (33.3)	N/A	46 (50.5)
QID	11 (25.5)	3 (12)	4 (40)	6 (60)	2 (66.7)	N/A	26 (28.6)
5×/day	5 (11.6)	4 (16)	1 (10)	2 (20)	0 (0)	N/A	12 (13.2)
6×/day	2 (4.7)	0 (0)	0 (0)	1 (10)	0 (0)	N/A	3 (3.3)
7×/day	1 (2.3)	0 (0)	0 (0)	0 (0)	0 (0)	N/A	1 (1.1)
8×/day	2 (4.7)	0 (0)	0 (0)	0 (0)	0 (0)	N/A	2 (2.2)
Time between arrival to C‐L order (h:mm)							
Mean (SD)	4:57 (3:24)	5:06 (4:46)	3:08 (1:43)	2:52 (1:55)	3:25 (3:41)	N/A	4:31 (3:37)
Median	4:04	4:48	3:08	2:32	1:51	N/A	3:48
Time between arrival to C‐L administration (h:mm)							
Mean (SD)	6:47 (4:15)	7:27 (5:38)	3:47 (2:09)	3:37 (1:58)	3:50 (3:34)	N/A	6:11 (4:29)
Median	5:52	6:43	3:55	3:15	2:41	N/A	5:18
Ordering specialty, *n* (%)							
Emergency Medicine	28 (65.1)	12 (48)	8 (80)	8 (80)	3 (100)	N/A	59 (64.8)
Internal Medicine	10 (23.3)	12 (48)	2 (20)	2 (20)	0 (0)	N/A	26 (28.6)
Neurology	4 (9.3)	Not available at this site	Not available at this site	Not available at this site	Not available at this site	Not available at this site	4 (4.4)
General Surgery	1 (2.3)	1 (4)	0 (0)	0 (0)	Not available at this site	Not available at this site	2 (2.2)
ED length of stay (h:mm)							
Mean (SD)	16:20 (11:12)	22:30 (15:51)	6:21 (1:36)	6:19 (2:06)	5:25 (2:51)	N/A	15:28 (12:44)
Median	12:17	21:52	6:03	7:28	5:30	N/A	9:28

Across all sites, the most commonly prescribed scheduling frequencies for C‐L were TID, QID, and 5 times daily. Of the 91 encounters in which C‐L was administered, 23 (25.3%) included existent outpatient prescriptions with documented administration times. In the remaining 68 encounters (74.7%), dosing times were inferred based on the standard EMR scheduling parameters (Table A1).

Of the 282 ED encounters, 12 (4.3%) met the idealized definition of timely C‐L administration, defined as dosing within 30 min of the scheduled time. Using the health system's broader medication administration standard, 18 encounters (6.4%) received C‐L within a two‐hour window (up to one hour before or one hour after the scheduled dose). Among the 91 encounters in which C‐L was ordered, this corresponded to 13.2% meeting the ideal timing definition and 19.8% meeting the system‐defined standard.

Among the remaining encounters in which C‐L was administered but did not meet the ideal timing definition (*n* = 79), administration occurred 1–4 h from the scheduled dose in 36 encounters (39.6%), 4–8 h in 24 encounters (26.4%), and more than 12 h before or after the scheduled time in 3 encounters (3.3%). All three encounters with delays exceeding 12 h involved patients who were admitted but boarding in the ED.

In the subset of 23 encounters with explicitly documented outpatient dosing times, 3 encounters (13.0%) met the ideal definition of timely administration, and 5 encounters (21.7%) met the health system definition of timely administration within a two‐hour window.

**FIGURE 1:**
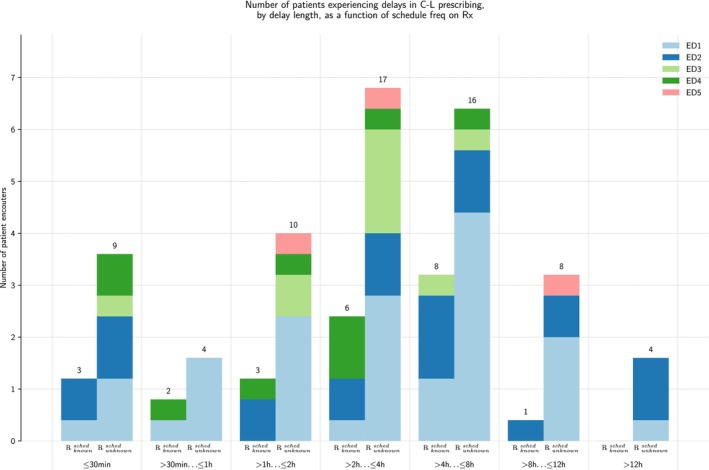
Number of patients experiencing delays in C‐L prescribing, by delay length, as a function of schedule frequency on prescription (Rx).

Figure 1 illustrates the timing of C‐L administration across all ED sites for both patients with documented outpatient dosing schedules and those whose schedules were inferred. Regardless of scheduling documentation status or ED location, the majority of patients experienced substantial delays in medication administration. A higher proportion of patients received a timely dose of their home Parkinson's medication at ED2 (8.2%) as compared to ED1 (4.1%), and ED4 (4.4%).

## 4 Discussion

The prevalence of PD in the United States is approximately 1 million, with incidence increasing substantially with age [9]. No therapy has been proven to slow disease progression or provide a cure; thus, treatment focuses on modulating bothersome symptoms and improving quality of life. Oral C‐L formulations remain the mainstay for controlling motor symptoms such as bradykinesia, rigidity, and tremor [10]. Scheduled administration of C‐L is recommended to maximize symptomatic benefit and maintain consistent motor control, thereby minimizing fluctuations in motor symptoms [10]. Adherence to dosing schedules is associated with improved symptom stability and reduced motor complications [10].

Medication reconciliation (the process of obtaining and verifying a complete and accurate list of a patient's medications) is a recognized challenge in the ED environment [11, 12, 13, 14]. Despite growing recognition of medication reconciliation as a core component of geriatric emergency care [13], the specific issue of time‐sensitive antiparkinsonian medication administration in the ED has not been well studied. Patients with PD often have complex medication regimens as part of their broader polypharmacy, yet the time‐dependent nature of dopaminergic therapy adds a unique urgency that distinguishes it from other chronic medications.

Our review of ED encounters over one year revealed that nearly 70% of patients with PD did not receive their antiparkinsonian medication while in the ED. A greater proportion of patients at the larger, non‐rural EDs (ED1 and ED2) received their antiparkinsonian medication as compared to those at rural community and critical access sites (EDs 3–6). Given that most C‐L prescriptions were scheduled for TID, QID, or 5 times daily dosing, and the mean ED LOS was nearly 9 h, most patients would have been due for at least one dose during their visit. Timely administration of C‐L occurred in only a small minority of ED encounters, occurring in just 4.3% of encounters by the idealized definition and 6.4% by the more permissive health system‐defined standard.

Our study demonstrates a lack of timely C‐L administration in the ED. Multiple factors likely contribute to both non‐ordering and delayed administration of C‐L in the ED. Emergency clinicians manage competing priorities and frequent task‐switching, which may lead to de‐prioritization of chronic home medications. In addition, under‐recognition of the time‐sensitive nature of antiparkinsonian therapy, uncertainty regarding patients' home dosing regimens, and challenges with medication reconciliation in the acute care setting may further contribute to omissions and delays [11]. Given the prolonged ED LOS observed in our study (mean 8 h 53 min) and the frequency of ED boarding, patients are at significant risk of missing multiple scheduled doses during their ED encounter. A systematic solution is therefore needed to ensure timely administration of antiparkinsonian medications in the ED. The next phase of this study will evaluate whether implementation of a BPA increases rates of timely C‐L administration in the ED.

## 5 Limitations

This study has several limitations. The chart review did not capture circumstances in which ordering or administering C‐L would have been contraindicated (such as if the patient was NPO), nor instances of patients who had self‐administered their home medication. As a result, the frequency of missed or delayed home antiparkinsonian medication administration may be overestimated. Evaluation of administration timeliness was also limited by the lack of specificity in outpatient prescriptions, with only 25.3% including explicit administration times. For the remaining prescriptions, scheduled dosing times were inferred based on default frequency definitions in our EMR. This approach may not reflect patients' individualized home regimens and could affect the assessment of administration timeliness. This study was not designed to measure downstream clinical outcomes. Although prior literature suggests that missed or delayed antiparkinsonian dosing may contribute to adverse effects, we cannot directly attribute clinical harm to the medication omissions or delays observed in this cohort. Future studies should evaluate associations between ED medication administration and outcomes such as delirium, mobility, falls, length of stay, and disposition. Finally, the generalizability of this study's results is limited by the lack of demographic diversity, with 99.3% of patients identifying as White. In addition, this study was conducted within a single health system which may limit the applicability of the findings to other settings with different workflows and EMR systems.

## 6 Conclusion

Emergency clinicians are not trained to prioritize chronic disease management and may not routinely order home medications in the acute care setting. For patients with PD, timely administration of antiparkinsonian medication is critical, as delayed or missed doses can potentially lead to significant morbidity and mortality. Our study revealed that less than one‐third of patients with PD received their home antiparkinsonian medication in the ED, and fewer than 10% of patients received it on time. Targeted interventions to ensure timely medication administration are needed to prevent iatrogenic harm in this vulnerable population. Beyond the implications for patients with PD, strategies to improve home medication ordering and administration in the ED may be broadly applicable to other disease processes and outpatient treatment regimens that patients may be missing in the ED. Future studies are needed to directly measure the clinical consequences of missed or delayed dosing in the ED.

## Author Contributions

N.M.E. contributed to the study concept and design, acquisition of data, analysis and interpretation of the data, drafting of the manuscript, and critical revision of the manuscript for intellectual content. E.M.L. contributed to the analysis and interpretation of the data, drafting of the manuscript, and critical revision of the manuscript for intellectual content. C.T.‐T. contributed to the critical revision of the manuscript for intellectual content and statistical expertise.

## Conflicts of Interest

The authors declare no conflicts of interest.

## Data Availability

The data that support the findings of this study are available from the corresponding author upon reasonable request.

## Appendix

**TABLE A1:** Dosing administration schedules with corresponding times based on EMR defaults.

Frequency	Inferred times medication due
BID	9:00, 21:00
TID	9:00, 14:00, 21:00
QID	8:00, 12:00, 17:00, 21:00
5‐times daily	7:00, 11:00, 15:00, 18:00, 21:00
6‐times daily	6:00, 9:00, 12:00, 15:00, 18:00, 21:00
7‐times daily	6:00, 9:00, 11:00, 14:00, 17:00, 19:00, 21:00
8‐times daily	6:00, 8:00, 10:00, 12:00, 14:00, 16:00, 18:00, 20:00
